# A Comparison of Percutaneous Endoscopic Lumbar Discectomy and Open Lumbar Microdiscectomy for Lumbar Disc Herniation in the Korean: A Meta-Analysis

**DOI:** 10.1155/2018/9073460

**Published:** 2018-08-07

**Authors:** Manyoung Kim, Sol Lee, Hyeun-Sung Kim, Sangyoon Park, Sang-Yeup Shim, Dong-Ju Lim

**Affiliations:** ^1^Department of Orthopedic Surgery, Gangnam Nanoori Hospital, 731 Eonju-ro, Gangnam-gu, Seoul 06048, Republic of Korea; ^2^Department of Health Policy and Management, Korea University, 145 Anam-ro, Seongbuk-gu, Seoul 02841, Republic of Korea; ^3^Department of Neurosurgery, Gangnam Nanoori Hospital, 731 Eonju-ro, Gangnam-gu, Seoul 06048, Republic of Korea; ^4^Department of Orthopedic Surgery, Sanggye Paik Hospital, Inje University College of Medicine, 1342 Dongil-ro, Nowon-gu, Seoul 01757, Republic of Korea

## Abstract

**Background:**

Among the surgical methods for lumbar disc herniation, open lumbar microdiscectomy is considered the gold standard. Recently, percutaneous endoscopic lumbar discectomy is also commonly performed for lumbar disc herniation for its various strong points.

**Objectives:**

The present study aims to examine whether percutaneous endoscopic lumbar discectomy and open lumbar microdiscectomy show better results as surgical treatments for lumbar disc herniation in the Korean population.

**Methods:**

In the present meta-analysis, papers on Korean patients who underwent open lumbar microdiscectomy and percutaneous endoscopic lumbar discectomy were searched, both of which are surgical methods to treat lumbar disc herniation. The papers from 1973, when percutaneous endoscopic lumbar discectomy was first introduced, to March 2018 were searched at the databases of MEDLINE, EMBASE, PubMed, and Cochrane Library.

**Results:**

Seven papers with 1254 patients were selected. A comparison study revealed that percutaneous endoscopic lumbar discectomy had significantly better results than open lumbar microdiscectomy in the visual analogue pain scale at the final follow-up (leg: mean difference [MD]=-0.35; 95% confidence interval [CI]=-0.61, -0.09; p=0.009; back: MD=-0.79; 95% confidence interval [CI]=-1.42, -0.17; p=0.01), Oswestry Disability Index (MD=-2.12; 95% CI=-4.25, 0.01; p=0.05), operation time (MD=-23.06; 95% CI=-32.42, -13.70; p<0.00001), and hospital stay (MD=-4.64; 95% CI=-6.37, -2.90; p<0.00001). There were no statistical differences in the MacNab classification (odds ratio [OR]=1.02; 95% CI=0.71, 1.49; p=0.90), complication rate (OR=0.72; 95% CI=0.20, 2.62; p=0.62), recurrence rate (OR=0.83; 95% CI=0.50, 1.38; p=0.47), and reoperation rate (OR=1.45; 95% CI=0.89, 2.35; p=0.13).

**Limitations:**

All 7 papers used for the meta-analysis were non-RCTs. Some differences (type of surgery (primary or revisional), treatment options before the operation, follow-up period, etc.) existed depending on the selected paper, and the sample size was small as well.

**Conclusion:**

While percutaneous endoscopic lumbar discectomy showed better results than open lumbar microdiscectomy in some items, open lumbar microdiscectomy still showed good clinical results, and it is therefore reckoned that a randomized controlled trial with a large sample size would be required in the future to compare these two surgical methods.

## 1. Introduction

Among the surgical methods for lumbar disc herniation, open lumbar microdiscectomy (OLD) is considered the gold standard [1]. Lumbar disc herniation is a common cause of low back pain and radiating pain to the lower extremities [2] and conservative therapy can improve the symptoms in most cases. In 10-20% of these cases, pain continues despite conservative therapy, and surgical treatment is considered [3]. While OLD can rarely cause scar tissues around nerves, damage to facet joints, and lumbar instability after the operation, it is widely performed as it shows good clinical results [4–7].

Recently, percutaneous endoscopic lumbar discectomy (PELD) is also commonly performed for lumbar disc herniation for its various strong points compared to OLD such as surgery under local anesthesia, less damage to surrounding muscles and bone structures, and fast patient recovery [8–12]. Indications were limited depending on the location and progression of lesions in early days [13, 14], but lately these limitations have been overcome owing to advances in technology and tools [9–12].

Nevertheless, it has not been clearly confirmed whether PELD, which had good results recently, is better than OLD, the gold standard, in Korean patients.

The purpose of this study is to determine through a meta-analysis whether PELD or OLD has better results as a surgical treatment for lumbar disc herniation in the Korean population.

## 2. Methods

### 2.1. Literature Search Strategy

Relevant studies were searched in MEDLINE, EMBASE, PubMed, and Cochrane Library. Retrieval time was from 1973, when PELD was first introduced, to March 2018. The papers were extracted using search keywords such as “lumbar disc herniation,” “microdiscectomy,” “percutaneous endoscopic lumbar discectomy,” “intervertebral disc displacement,” “transforaminal lumbar discectomy,” “minimally invasive discectomy,” and “interlaminar discectomy”; the researcher extracted only those studies conducted on humans, which were written in English.

### 2.2. Inclusion Criteria and Exclusion Criteria

Two authors (M Kim and S Lee) identified the titles and abstracts or both and summarized the data from the selected articles. The searched papers were selected based on the following criteria: (1) those which were either randomized or nonrandomized controlled trials, (2) those that had at least one significant result on Korean patients, and (3) those on patients who underwent PELD or OLD for lumbar disc herniation. The papers on those who had a combined surgery and lesions in more than one area and case reports, letters, and comments were excluded.

### 2.3. Data Extraction

The following data were extracted from the papers collected by two of the authors (M Kim and S Lee): (a) basic information such as the type of trial, follow-up period, type of surgery, sample size, and patient age and sex and (b) clinical results such as the visual analogue pain scale (VAS) score (leg and back), complication rate, recurrence rate, reoperation rate, hospital stay, operation time, MacNab classification, and Oswestry Disability Index (ODI).

### 2.4. Quality Assessment

All 7 collected papers were nonrandomized clinical trials, and the Newcastle-Ottawa Quality Assessment Scale (NOQAS) was used for quality assessment. Out of a possible 9 items, 3 of selection, comparability, and exposure or outcome account for 4, 2, and 3 points, respectively. Five points or more indicated a low risk of bias, while 4 or less indicated having a high risk of bias [15].

### 2.5. Statistical Analysis

The continuous variables (VAS, hospital stay, operation time, and ODI) were weighted with the number of patients, and the weighted average results were calculated. They were analyzed using standard deviations at a 95% confidence interval (CI). Meanwhile, the binary variables (complication rate, recurrence rate, reoperation rate, and MacNab score) were analyzed using the odds ratio (OR) at a 95% CI. I2 statistics were used to determine heterogeneity, and more than 50% was regarded as heterogeneous. The Review Manager software (version 5.3; The Cochrane Collaboration, Oxford, United Kingdom) was used as a statistical program for analysis.

## 3. Result

### 3.1. Identification of Relevant Studies

**Figure 1:**
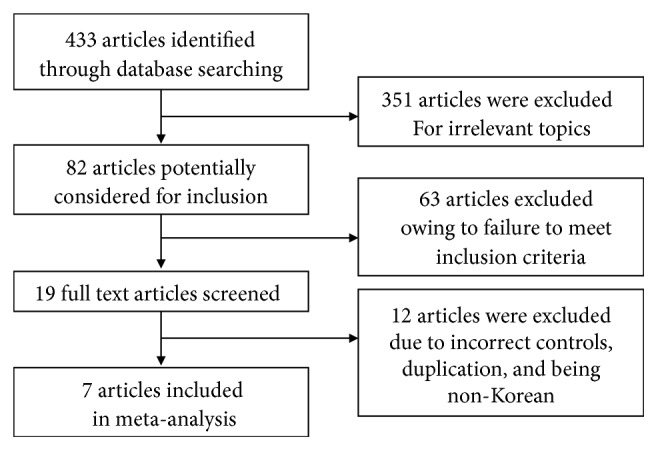
Flow diagram detailing study inclusion.

A total of 433 papers were searched, and 426 of them, which did not meet the selection criteria, were excluded.  Figure 1 illustrates how the papers were selected, and the final 7 papers satisfied the inclusion criteria and were included in this study's analysis [11, 16–21].

### 3.2. Study Characteristics and Quality Assessment

The basic characteristics of the selected papers are presented in Table 1. All of the 7 selected papers were nonrandomized retrospective studies. The quality assessment results are provided in Table 2, and, except for one paper that scored 4 points in NOQAS, the other papers scored 5–7 points and showed good results in the quality assessment.

### 3.3. Meta-Analysis Results

#### 3.3.1. VAS Score at the Final Follow-Up

**Figure 2:**
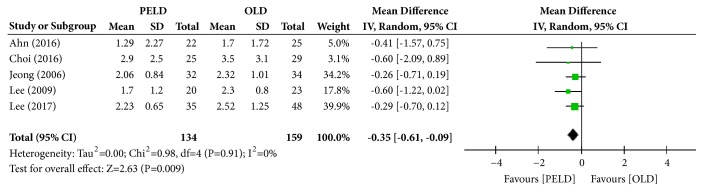
Forest plot of comparison: PELD versus OLD; outcome: 1-1 for VAS (leg), final follow-up.

Among the 7 papers, 5 presented the results of the VAS (leg), and 293 subjects were included in the analysis: 134 in the PELD group and 159 in the OLD group. The PELD group's average VAS was 2.04, while that of the OLD group was 2.47. The PELD group showed a significantly lower average VAS (leg) at the final follow-up than the OLD group (mean difference [MD]=-0.35; 95% CI=-0.61, -0.09; p=0.009) (Figure 2). No heterogeneity existed between individual studies included in the analysis (I^2^=0%, p=0.91).

**Figure 3:**
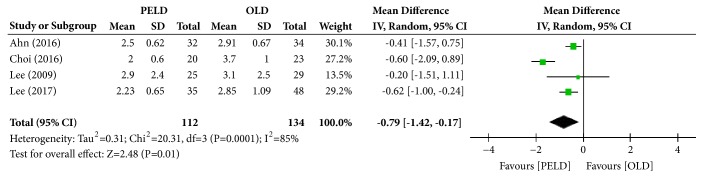
Forest plot of comparison: PELD versus OLD; outcome: 1-2 for VAS (back), final follow-up.

Among the 7 papers, 4 presented the results of the Visual VAS (back), and 246 subjects were included in the analysis: 112 in the PELD group and 134 in the OLD group. The PELD group's average VAS (back) was 2.40, while that of the OLD group was 3.14. The PELD group showed a significantly lower average VAS (back) at the final follow-up than the OLD group (MD=-0.79; 95% CI=-1.42, -0.17; p=0.01) (Figure 3). Heterogeneity existed between individual studies included in the analysis (I^2^=85%, p=0.0001).

#### 3.3.2. MacNab Classification at the Final Follow-Up

**Figure 4:**
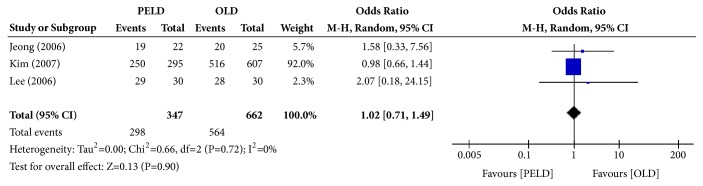
Forest plot of comparison: PELD versus OLD, outcome: 2 for MacNab classification (success rate).

Among the 7 papers, 3 presented the results of the MacNab score (success rate), and 1,009 subjects were included in the analysis: 347 in the PELD group and 662 in the OLD group. Those who answered with excellent or good were defined as successful, and 298 among the 347 subjects in the PELD group answered with successful in the MacNab criteria. Among the 662 subjects in the OLD group, 564 answered with successful. There were no significant differences in the average MacNab score (success rate) between the PELD and OLD groups (odds ratio [OR]=1.02; 95% CI=0.71, 1.49; p=0.90) (Figure 4). There was no heterogeneity between individual studies included in the analysis (I^2^=0%, p=0.72).

#### 3.3.3. ODI

**Figure 5:**
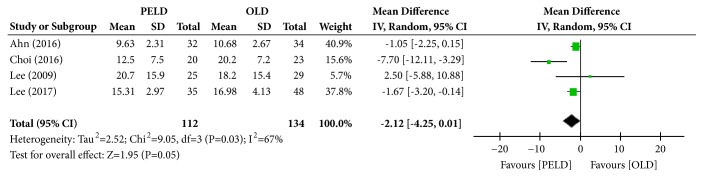
Forest plot of comparison: PELD versus OLD; outcome: 3 for ODI, final follow-up.

Among the 7 papers, 4 presented the results of the ODI, and 246 subjects were included in the analysis: 112 in the PELD group and 134 in the OLD group. The PELD group's average ODI was 14.54%, while that of the OLD group was 16.52%. The PELD group showed a significantly lower average ODI at the final follow-up than the OLD group (MD=-2.12; 95% CI=-4.25, 0.01; p=0.05) (Figure 5). Heterogeneity existed between individual studies included in the analysis (I^2^=67%, p=0.03).

#### 3.3.4. Complication Rate

**Figure 6:**
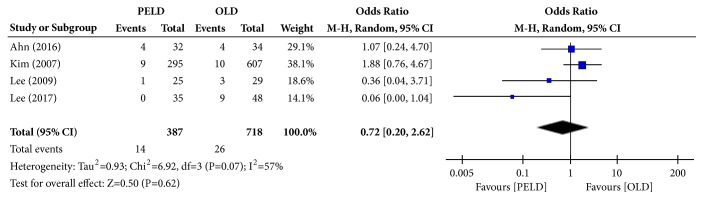
Forest plot of comparison: PELD versus OLD; outcome: 4 for complication rate.

Among the 7 papers, 4 presented the results of the complication rate, and 1,105 subjects were included in the analysis: 387 in the PELD group and 718 in the OLD group. Fourteen subjects in the PELD group and 26 subjects in the OLD group had complications. There were no significant differences in the complication rate between the PELD and OLD groups (OR=0.72; 95% CI=0.20, 2.62; p=0.62) (Figure 6). Heterogeneity existed between individual studies included in the analysis (I^2^=57%, p=0.07).

#### 3.3.5. Recurrence Rate

**Figure 7:**
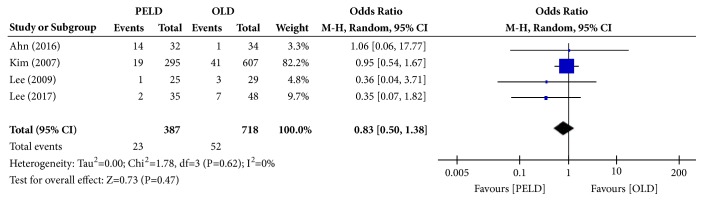
Forest plot of comparison: PELD versus OLD; outcome: 5 for recurrence rate.

Among the 7 papers, 4 presented the results of the recurrence rate, and 1,105 subjects were included in the analysis: 387 in the PELD group and 718 in the OLD group. Twenty-three subjects in the PELD group and 52 in the OLD group had recurrence. There were no statistically significant differences in the recurrence rate between the PELD and OLD groups (OR=0.83; 95% CI=0.50, 1.38; p=0.47) (Figure 7). There was no heterogeneity between individual studies included in the analysis (I^2^=0%, p=0.62).

#### 3.3.6. Reoperation Rate

**Figure 8:**
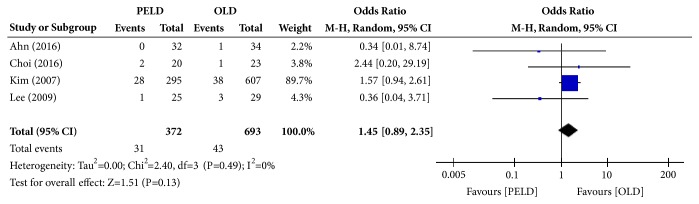
Forest plot of comparison: PELD versus OLD; outcome: 6 for reoperation rate.

Among the 7 papers, 4 presented the results of the reoperation rate, and 1,065 subjects were included in the analysis: 372 in the PELD group and 693 in the OLD group. Thirty-one subjects in the PELD group and 43 subjects in the OLD group had reoperation. There were no significant differences in the reoperation rate between the PELD and OLD groups (OR=1.45; 95% CI=0.89, 2.35; p=0.13) (Figure 8). There was no heterogeneity between individual studies included in the analysis (I^2^=0%, p=0.49).

#### 3.3.7. Operation Time

**Figure 9:**
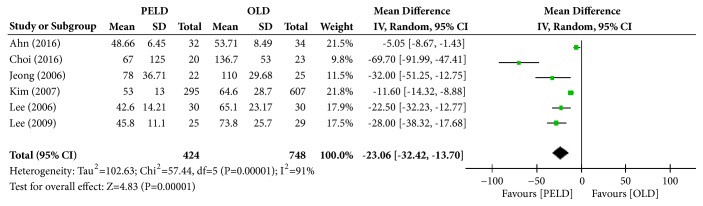
Forest plot of comparison: PELD versus OLD; outcome: 7 for operation (minute).

Among the 7 papers, 6 presented the results of operation time, and 1,172 subjects were included in the analysis: 424 in the PELD group and 748 in the OLD group. The PELD group's average operation time was 55.84 min, and that of the OLD group was 83.99 min. The PELD group's average operation time was significantly shorter than that of the OLD group (MD=-23.06; 95% CI=-32.42, -13.70; p<0.00001) (Figure 9). Heterogeneity existed between individual studies included in the analysis (I^2^=91%, p<0.00001).

#### 3.3.8. Hospital Stay

**Figure 10:**
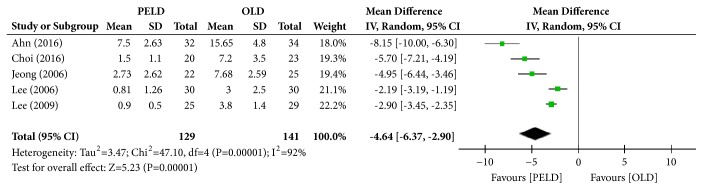
Forest plot of comparison: PELD versus OLD; outcome: 8 for hospital stay (days).

Among the 7 papers, 5 presented the results of hospital stay, and 270 subjects were included in the analysis: 129 in the PELD group and 141 in the OLD group. The PELD group's average hospital stay was 2.69 days, and that of the OLD group was 7.47 days. The PELD group's average hospital stay was significantly shorter than that of the OLD group (MD=-4.64; 95% CI=-6.37, -2.90; p<0.00001) (Figure 10). Heterogeneity existed between individual studies included in the analysis (I^2^=92%, p<0.00001).

## 4. Discussion

In general, OLD has been mostly performed as a surgical treatment for lumbar disc herniation. This technique could possibly lead to lumbar instability and iatrogenic injury as it requires the removal of some posterior structures such as lamina, ligament flavum, and facet joints, dissection of muscles near the spine, and pulling of nerve branches [22, 23]. In response, PELD, which had relatively smaller loss of posterior structures and faster early recovery, was introduced by Kambin and Gellman [24] and is recently used widely for its strength where it can be performed under local anesthesia [25]. However, PELD also has its downsides; that is, it insufficiently removes the disc, has a high recurrence rate, and requires a certain period of time to develop skill proficiency [26–28], and therefore it calls for a comparison of these two surgical methods for their stability and effect.

Recently, a meta-analysis, which compared OLD and PELD as surgical treatments for lumbar disc herniation, reported two cases in 2016 [29, 30]. Each meta-analysis was performed by extracting data from 7 papers; the papers were limited to those published after 2000, when endoscopic technology and tools were advanced, and as a result each one selected 5 and 6 papers. The studies conducted on Koreans patients accounted for a majority with 3 and 4 papers. Considering this could work as a bias, this study performed a meta-analysis only on those conducted on Korean patients.

In the present study, PELD showed statistically significantly better results than OLD in the VAS score (of both leg and back) at the final follow-up, ODI, operation time, and hospital stay. We believe it is especially meaningful that contrary to previous meta-analysis studies [29, 30] PELD showed better results in the VAS score as it is the primary outcome of the surgery. In previous meta-analysis studies [29, 30], PELD showed better results in operation time and hospital stay in both and this study, whereas PELD showed better results in the ODI in one of the two previous meta-analysis studies [29] and this study. PELD showed statistically significantly better results in more items in this study than in previous studies. It is because endoscopic surgery has less damage to muscles and structures around the spine as well-known [31, 32]. In addition, we believe that the papers included in this study are relatively recent researches with the enough development of endoscopic instruments and proficiency of endoscopic skills.

The MacNab classification was recorded in only 3 among the 7 papers, and there were no significant differences between the two surgical methods. At the final follow-up, both surgical methods showed successful results. So, we have determined that both methods are effective.

In this study, there were no significant differences in reoperation and recurrence rate. There were two previous reports stating that there were no differences between the two surgical methods [14, 33] and there was another report that PELD had more recurrence and reoperation rate [26]. Usually, in such cases, there was a remaining disc piece or it was accompanied by stenosis due to reoperation and recurrence [34], and it would be important to determine appropriate indications as well as develop the proficiency of surgical skills.

Complications included infection, spinal cord injury, cerebrospinal fluid leak, damage to nerve roots, and postoperative sensory abnormalities [11, 16, 19, 20]. A previous report stated that PELD had fewer complications, thanks to the development of tools such as the camera system [35], but another reported that it would do more damage to the spinal cord and nerve roots due to a lack of depth [36]. In this study, each paper showed different results, and there were no statistically significant differences.

This study has some limitations. All 7 papers used for the meta-analysis were nonrandomized trials, and there was selection bias as a result. The quality of these trials was also fairly high. In addition, clinical heterogeneity existed in this study. Type of surgery (primary or revisional), surgical indications, treatment options before the operation, and follow-up period during the symptom period varied depending on the selected paper, and the sample size was small as well. Particularly revision surgery may demand different approach-related surgical technique and more operation time. They may be subject to more complications that do not exist during the initial surgery. Finally, a physician's proficiency makes a huge difference in PELD, and it is believed that the difference in physicians in each paper would have worked as a bias.

## 5. Conclusion

This meta-analysis found that PELD had significantly better results than OLD in the VAS score, ODI, operation time, and hospital stay as a surgical treatment for lumbar disc herniation in the Korean population. Nevertheless, OLD still showed good clinical results, and therefore a randomized controlled study with a large sample size would be required in the future to compare these two surgical methods.

## Figures and Tables

**Table 1:** Baseline characteristics of included studies.

Author and year	Study design	Number of patients (male/female)		Patient age (years)		Follow-up time (month)	
		PELD	OLD	PELD	OLD	PELD	OLD
Jeong (2006)	Non-randomized retrospective comparative	22(14/8)	25(16/9)	56.45±10.89	56±9.12	12	12

Lee (2006)	Non-randomized retrospective comparative	30(22/8)	30(22/8)	39.3(22-67)	39.6(20-64)	38.2(32-45)	36.8(35-42)

Kim (2007)	Non-randomized retrospective comparative	295(188/107)	607(392/215)	34.9(13-83)	44.4(17-80)	23.6(18-36)	23.6(18-36)

Lee (2009)	Non-randomized retrospective comparative	25(16/9)	29(22/7)	42.0±11.4	47.7±12.2	34.0±4.4	34.3±4.6

Ahn (2016)	retrospective cohort	32(32/0)	34(34/0)	22.41±1.68	22.18±1.51	13.69±1.26	13.41±1.02

Choi (2016)	retrospective cohort	20(14/6)	23(13/10)	33.9±11.1	38±11.6	27.5±5.7	27.5±5.7

Lee (2017)	Non-randomized retrospective comparative	35(25/10)	48(30/18)	50.20±12.87	50.13±11.56	24.17±11.83	23.65±7.94

**Table 2:** Risk of bias assessment of the nonrandomized studies.

Studies	Selection	Comparability	Exposure	Total Quality score
Jeong (2006)	2	2	1	5
Lee (2006)	2	2	3	7
Kim (2007)	2	1	1	4
Lee (2009)	2	2	1	5
Lee (2017)	2	2	2	6
Ahn (2016)	2	2	2	6
Choi (2016)	2	2	1	5
